# Separated Surgical Instrument During the Extraction of a Third Molar: A Case Report

**DOI:** 10.7759/cureus.91189

**Published:** 2025-08-28

**Authors:** Abdulaziz A Mahdi, Abdullah I Alkharji, Safa A Alburayh, Bader A Fatani, Osama A Alharbi

**Affiliations:** 1 Maxillofacial Surgery Department, College of Dentistry, King Saud University, Riyadh, SAU; 2 Maxillofacial Surgery Service, Security Forces Hospital, Riyadh, SAU; 3 Maxillofacial Surgery Department, College of Dentistry, Imam Abdulrahman bin Faisal University, Dammam, SAU

**Keywords:** foreign body, iatrogenic complication, mandibular third molar, nerve preservation, oral and maxillofacial surgery, separated bur, separated surgical instrument, third molar, tooth extraction

## Abstract

Third molar extraction is among the most frequently performed procedures in oral surgery. The indications for extraction, such as dental caries, orthodontic considerations, periodontal disease, or associated pathology, vary between patients; consequently, the complications encountered can differ significantly. In this case report, we describe an uncommon complication involving the separation of a surgical bur during the extraction of a mandibular third molar. The patient underwent careful monitoring for three years, after which the retained instrument fragment was successfully retrieved under local anesthesia without any permanent nerve damage or additional complications. Careful use of sharp, well-maintained surgical bur and prompt replacement of dull burs can prevent complications such as instrument separation, while advanced imaging radiographs and risk-based planning guide safe retrieval of retained fragments.

## Introduction

Postoperative sequelae (a logical consequence of a procedure), such as swelling, pain, and bleeding, are considered common and expected outcomes following the surgical extraction of third molars. Nevertheless, various complications (an unexpected, untoward event) can arise either intraoperatively or postoperatively [1]. Such complications include infection, injury to adjacent vital anatomical structures, displacement of teeth or root fragments into adjacent spaces, separation of surgical instruments, and fractures of the mandibular or maxillary bone.

The most common causes of instrument separation or breakage are using dull or corroded instruments, inappropriate use, the physical properties of the surgical instrument, possible manufacturing defects, excessive cutting depth, and pressure with lateral force [2]. Although bur separation during third molar extraction has been documented in the literature, no data currently exist regarding its exact prevalence. The ideal approach to managing complications is their prevention through careful preoperative planning, proper operative technique, and the appropriate selection and maintenance of surgical instruments.

There are various approaches to accessing and facilitating the retrieval of a separated instrument [2], depending on factors such as the size of the separated instrument, accessibility limitations, anatomical considerations, and the severity of symptoms [3]. In general, a surgeon should attempt to retrieve a separated instrument immediately when the incident occurs. In most cases, the separated instrument can be retrieved right away, especially when it remains visible. Retrieval is typically straightforward under these circumstances; however, if the separated instrument migrates deeper into surrounding tissues and is no longer visible, locating and retrieving it becomes significantly more difficult. The separated instrument should be retrieved as soon as possible to prevent migration into adjacent tissues. Prompt retrieval is essential to avoid potential complications such as infection, risk of swallowing, or aspiration. Moreover, if the separated instrument is small, retrieval may require extensive surgery, which can cause secondary injury [2].

The decision to operate on a patient, like many other surgical decisions, often falls within a discretionary gray zone where the optimal treatment option is not always clear. Knowing when to operate and when not to is considered an essential skill that every surgeon must master. Therefore, evaluating the likelihood of negative outcomes (risk) versus the likelihood of desirable outcomes (benefit) is a critical component of sound surgical decision-making [4]. 

In the present case, an unexpected intraoperative complication occurred, specifically, the breakage and separation of a surgical instrument during the extraction of a mandibular third molar. The initial retrieval attempt failed, and a decision was made to leave the separated instrument in situ based on a risk-benefit analysis. However, after three years, the decision was revisited due to changes in clinical circumstances, and the separated instrument was successfully retrieved. No postoperative complications were reported either after leaving the fragment in place, such as infection, or following its retrieval, such as paresthesia.

## Case presentation

**Figure 1:**
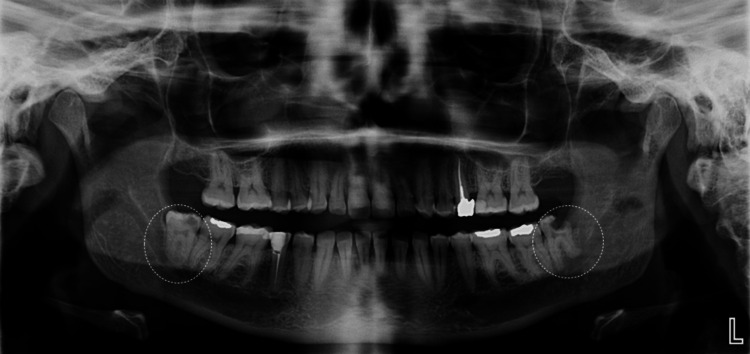
Preoperative panoramic radiograph (2021) showing mesial caries in tooth #48 and advanced decay in tooth #38.

A 43-year-old female patient with a history of palpitations, managed effectively with bisoprolol (5 mg daily), and no known allergies, presented to the emergency dental clinic in 2021, seeking extraction of both lower third molars. A thorough clinical examination and a panoramic radiograph were performed, confirming the presence of bilateral pathologic conditions involving these third molars. The preoperative panoramic radiograph showed a large mesial carious lesion in the lower right third molar (#48) and advanced decay affecting the crown of the lower left third molar (#38) (Figure 1).

The lower left third molar (#38) was necrotic on pulp testing, yet it was asymptomatic at the time of examination. By contrast, tooth #48 had extensive carious involvement extending toward the pulp chamber. The patient elected to prioritize the extraction of the lower right third molar (#48) first, based on her desire to preempt future episodes of severe dental pain on that side.

**Figure 2:**
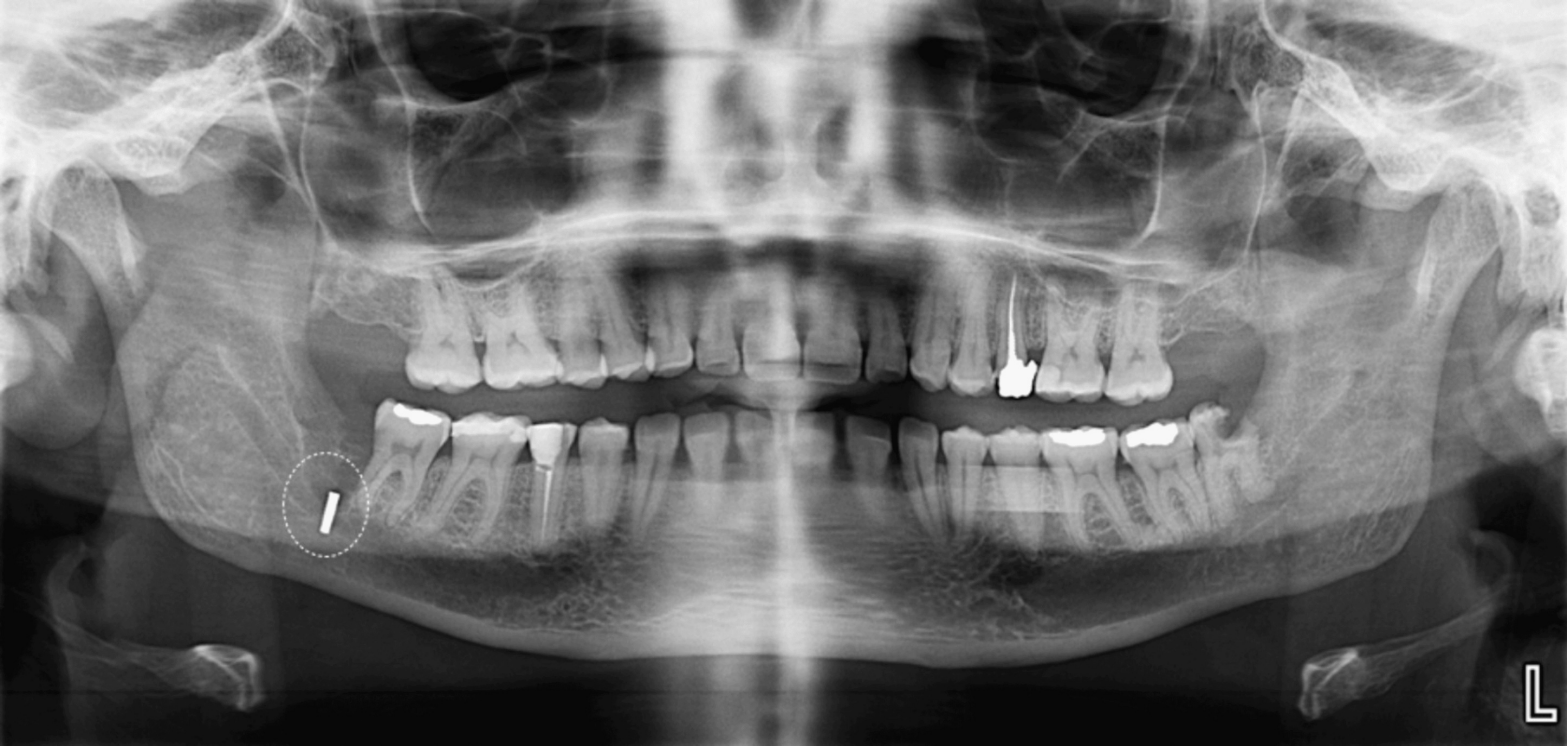
Panoramic radiograph (2022) taken immediately after bur separation.

Approximately one year after the initial visit, in 2022, the patient returned for definitive surgical management of the third molars. An Oral and Maxillofacial Surgery (OMFS) specialist evaluated her at that time, and the plan to extract tooth #48 first was confirmed. During the surgical extraction of tooth #48, a dull fissure bur fractured unexpectedly at the lingual aspect of the mandible. An immediate attempt was made to locate and retrieve the separated bur fragment intraoperatively; however, this retrieval proved unsuccessful due to the fragment’s close proximity to the lingual nerve, which raised significant concern about potential nerve injury. After careful intraoperative evaluation, the patient was informed about the incident, and a detailed risk-benefit analysis was conducted. It was concluded that the risk of causing permanent lingual nerve damage with aggressive immediate removal outweighed the potential benefit of retrieving the fragment at that moment. Therefore, the decision was made to leave the separated bur fragment in situ, with plans for periodic clinical and radiographic follow-up to monitor its position and ensure the patient’s safety. A panoramic radiograph taken immediately after the instrument separation (Figure 2) confirmed the presence of the bur fragment on the lingual side of the mandible adjacent to the #48 extraction site.

The patient’s recovery from the #48 extraction (with the bur fragment left in place) was initially uneventful. She was subsequently seen for extraction of the lower left third molar (#38), which was carried out successfully by the OMFS team without any complications or postoperative sequelae. Routine follow-up visits were scheduled and maintained after these procedures. At each visit, clinical examination and imaging were performed to ensure there were no changes in the fragment’s status or any new issues.

**Figure 3:**
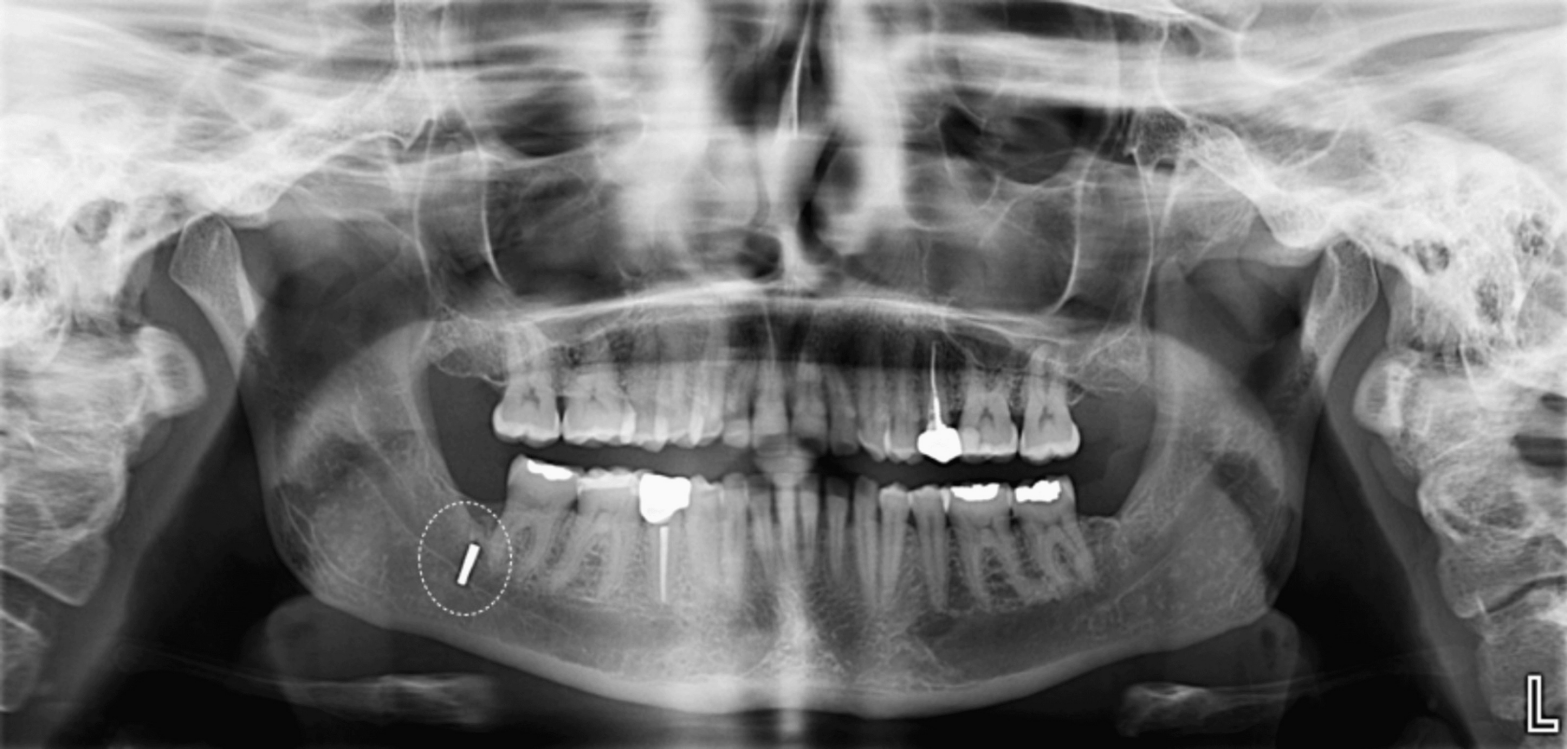
Panoramic radiograph at the three-year follow-up (2025).

Approximately three years later (in 2025), a follow-up panoramic radiograph (Figure 3) demonstrated that the separated bur fragment remained in the same position with no evidence of migration.

By this time, the patient had developed new neurological symptoms unrelated to her dental condition; she reported increasingly severe, generalized headaches of two days’ duration, accompanied by nausea and vomiting. Given this clinical presentation, the neurology team caring for the patient requested a magnetic resonance imaging (MRI) study of the brain to investigate the cause of her headaches. However, the retained metallic bur fragment posed a safety concern for MRI due to the potential risks of heating, movement, or significant image artifact formation during scanning. In light of these considerations, the patient was referred back to the OMFS department in 2025 for evaluation and surgical removal of the retained bur fragment prior to undergoing the MRI.

**Figure 4:**
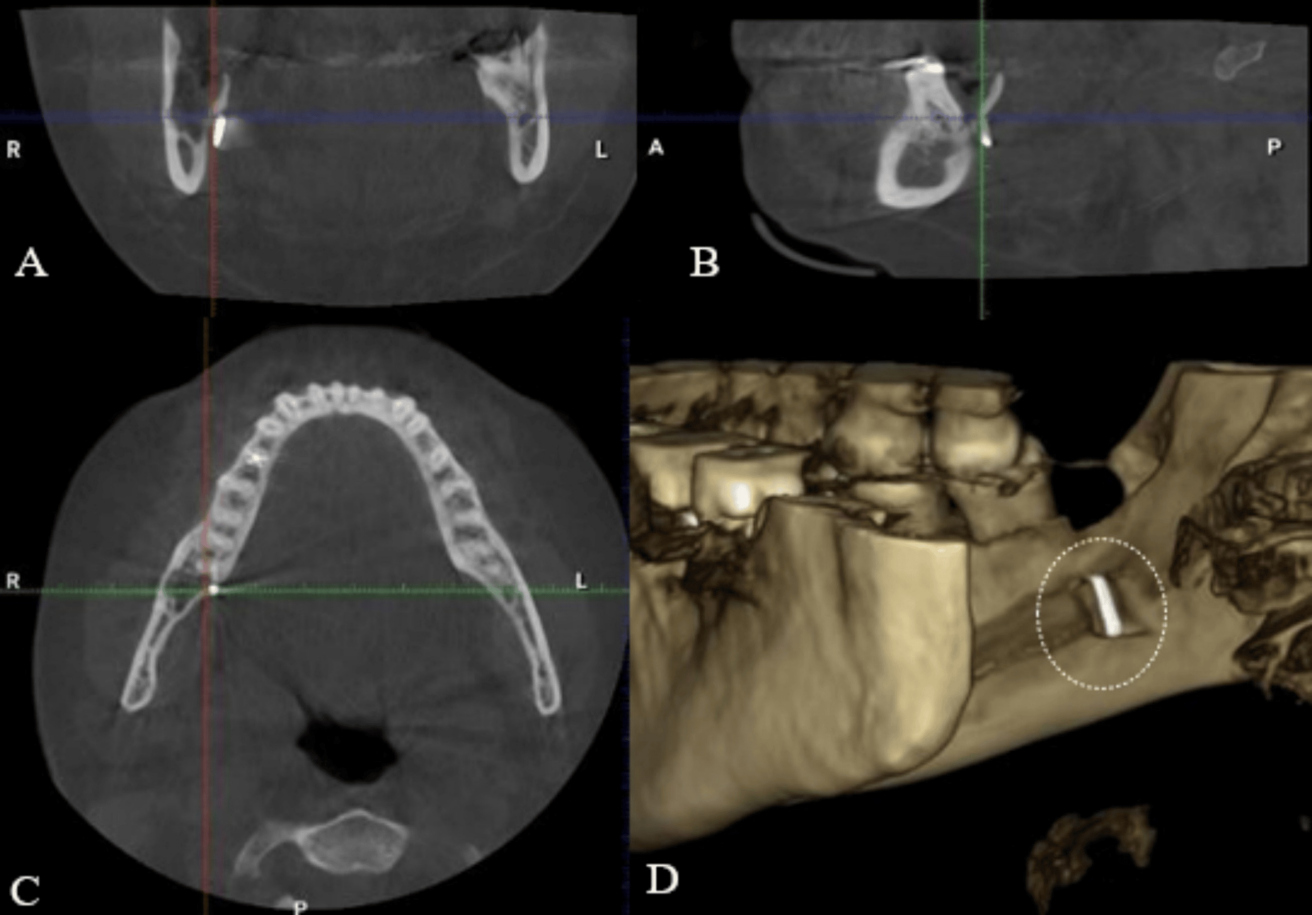
CBCT views to localizing the bur fragment lingual to tooth #48. (A) coronal, (B) sagittal, (C) axial, and (D) three-dimensional reconstruction CBCT: cone-beam computed tomography

A cone-beam computed tomography (CBCT) scan was obtained to precisely localize the fragment in relation to surrounding structures. The CBCT imaging clearly demonstrated that the separated fissure bur fragment was lodged on the lingual aspect of the mandibular right third molar region (#48), approximately at the level of the root apices of the extracted tooth. Importantly, the CBCT confirmed that the fragment’s position had remained stable and unchanged since the initial incident in 2022, with no migration or displacement observed (Figure 4).

After comprehensive counseling regarding the potential surgical risks, including the possibility of lingual nerve injury, informed consent was obtained for the retrieval of the bur fragment.

**Figure 5:**
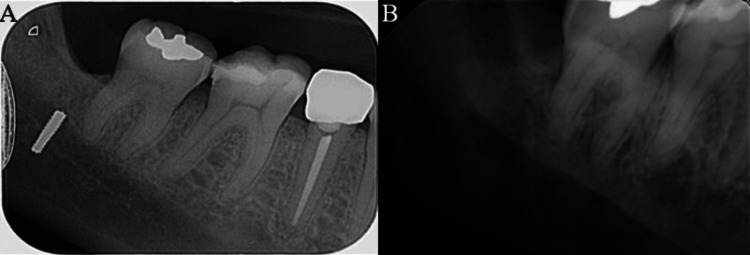
Comparison of (A) preoperative PA and (B) postoperative PA radiographs confirming bur removal. PA: periapical

The fragment retrieval procedure was performed in 2025 under local anesthesia. An inferior alveolar nerve block with a long buccal nerve block was administered, using two cartridges of 2% lidocaine with 1:80,000 epinephrine. The surgical site was approached through a full-thickness envelope flap on the lingual side of the mandible. The flap was carefully reflected using a #15 blade, and a number nine periosteal elevator was used to gently elevate the lingual periosteum and mucoperiosteal tissues. Blunt dissection was meticulously carried out on the lingual aspect to protect the integrity of the lingual nerve during the exploration. With optimal illumination and direct visualization, the bur fragment became palpable and was easily identified beneath the periosteal layer. External manual compression of the submandibular region with the surgeon’s left hand provided additional stabilization of the tissues. This maneuver facilitated the secure grasping of the fragment with hemostatic forceps, and the bur fragment was carefully removed from its sub-periosteal position. The underlying bony margins were smoothed with a bone file to remove any sharp edges and to promote proper healing of the bone and surrounding soft tissues. The surgical site was then irrigated and closed primarily with 3-0 Vicryl® resorbable sutures (Ethicon, Inc., Raritan, New Jersey, United States). Immediate postoperative confirmation of complete fragment retrieval was obtained using a periapical (PA) radiograph, which demonstrated successful removal of the metallic bur fragment (Figure 5).

At the two-week postoperative follow-up appointment, the surgical site had healed well, with no signs of infection or other complications. Notably, the patient exhibited no residual signs or symptoms of paresthesia in the distribution of the right lingual nerve; the right lower lip, chin, and tongue all had normal sensation. She reported that the transient numbness and tingling experienced immediately after the original 2022 surgery had fully resolved within a few weeks of that procedure. The patient was extremely relieved and satisfied with the outcome of the fragment retrieval. Following this successful intervention, she proceeded to undergo the prescribed MRI study, which was completed without incident. The patient continues to be followed at regular intervals, and to date, no further complications or adverse findings have been noted.

## Discussion

Dental pain has a profound negative impact on patients’ oral health-related quality of life, significantly influencing their overall well-being [5,6]. This clinical reality emphasizes the importance patients place on timely interventions to address symptomatic teeth. In the present case, although the lower left third molar (#38) was necrotic, it was asymptomatic; the patient’s decision to extract the contralateral third molar (#48) first was driven by a desire to prevent future pain on that side. The subsequent unexpected bur fracture and retention represented an uncommon iatrogenic complication. It is possible that the clinician perceived the case as a relatively simple and quick extraction procedure, and furthermore, may not have carefully inspected the bur for its condition (being dull) prior to use. Regarding the mechanism of this incident, it is not fully understood. The surgical team that performed the initial extraction differed from the team that later retrieved the fractured bur fragment, which limits the ability to establish the exact sequence of events. However, our theory is that the clinician performed adequate bone guttering and subsequent tooth sectioning, but the bur separated during tooth sectioning, and the application of excessive force during retrieval may have contributed to the displacement of the bur fragment.

Although the risk of lingual nerve injury is constant during both immediate and delayed retrieval procedures, a conservative approach was initially taken, leaving the fragment in situ, because the potential harm of surgical retrieval at that time outweighed the benefit. The patient’s postoperative follow-up over the next three years validated and confirmed this decision, as no issues arose from the retained fragment until the need for MRI clearance prompted re-evaluation. After three years, the patient consented to the procedure after understanding the risk-benefit ratio analysis, including undergoing an MRI to rule out any intracranial or head and neck malignancy. From a risk-benefit perspective, the potential for lingual nerve injury was considered acceptable when weighed against the need to exclude serious pathology such as brain or head and neck cancer. Effective management of such a complication requires balancing the urgency of fragment removal against the risk of collateral damage, particularly nerve injury.

Instrument breakage and the inadvertent retention of foreign objects during dental surgery are well-documented but infrequent occurrences. Matsuda et al. reported two cases of high-speed bur breakage and migration during third molar surgery [7]. Several factors can contribute to a bur fracture, including excessive lateral force during use, metal fatigue, manufacturing defects, or repeated use leading to wear and corrosion of the bur [7,8]. In the present case, the bur that fractured was noted to be dull, which likely resulted from multiple uses and inadequate replacement. Repeated sterilization cycles can degrade the structural integrity and cutting efficiency of dental burs [9,10]. Prolonged use of a bur without timely replacement increases the risk of overheating and metal fatigue, both of which can precipitate fracture. Additionally, corrosion from improper cleaning and drying can create microstructural weaknesses in rotary instruments [8]. To minimize these risks, surgeons are advised to adhere to instrument maintenance protocols, for example, inspection of burs before use, discarding any instruments that show signs of wear or corrosion, and avoiding excessive force or prolonged pressure when using rotary instruments on bone.

In the immediate aftermath of the bur separation in our case, the patient experienced transient numbness in the right lingual nerve distribution. This clinical finding was consistent with a mild, reversible nerve injury. Specifically, the temporary paresthesia corresponded to a Sunderland first-degree nerve injury, also known as neurapraxia [11]. Neurapraxia represents the mildest form of peripheral nerve injury and is typically caused by a transient conduction block due to focal demyelination at the site of injury. It commonly results from blunt trauma or mild compression of the nerve and manifests as sensory dysfunction, such as numbness, tingling, or altered sensation, that spontaneously resolves within days to weeks [12]. In our patient’s case, the sensory disturbance resolved completely within a few weeks after the initial surgery, confirming the neurapraxic nature of the injury and a return to normal nerve function. No permanent nerve damage occurred, which reinforced the initial decision to avoid aggressive fragment removal in 2022 when the risk to the nerve was highest.

The presence of the retained bur fragment became a concern when the patient required an MRI for unrelated neurologic symptoms. In general, metallic objects in a patient can pose hazards or compromise image quality during MRI scanning. Objects are classified for MRI safety as MRI-safe (no risk in MRI), MRI-unsafe (significant risk due to ferromagnetism), or MRI-conditional (safe only under certain conditions) [13]. Many dental instruments and burs are made from stainless steel alloys containing iron, which are ferromagnetic and thus MRI-unsafe [13]. Tungsten carbide, however, is the material used in the bur fragment involved in this case. Pure tungsten carbide lacks strong ferromagnetic properties. Nevertheless, variations in the alloy composition or minor impurities can confer a mild magnetic susceptibility to the bur fragment [13,14]. Even minimally magnetic objects can experience slight forces or heating in the MRI environment and can produce significant artifacts on the images [14]. In our patient’s situation, the fragment was likely tungsten carbide (a common bur tip material) and small in size, meaning the risk of projectile motion or heating was low; however, the potential for image distortion (artifact) was high. Consequently, removal of the retained bur fragment was deemed essential prior to the MRI in order to ensure patient safety and to allow accurate diagnostic imaging.

**Figure 6:**
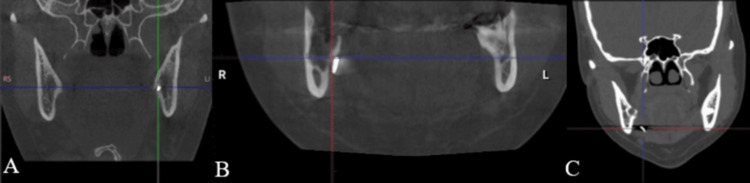
Illustration of three potential locations of a separated bur Image Credit for (A) and (C): Xing et al., 2025 [2]; licensed under a Creative Commons Attribution-NonCommercial-NoDerivatives 4.0 International License (CC BY-NC-ND 4.0) (https://creativecommons.org/licenses/by-nc-nd/4.0/) Image Credit for (B): Authors; from present case

Based on three-dimensional radiographic imaging such as CBCT, separated surgical burs can be classified anatomically into three distinct categories according to their location [2]. These are: (A) Type 1, located within the mandibular bone itself, (B) Type 2, positioned between the lingual alveolar cortical bone and the periosteum (as in the present case), and (C) Type 3, embedded within the soft tissues of the floor of the mouth (Figure 6).

Types 1 and 2 typically carry a favorable prognosis, as removal of the separated bur fragment is relatively straightforward due to its intraosseous or sub-periosteal position. In particular, Type 2 retrieval is generally less complicated and less time-consuming because the fragment lies in a relatively accessible sub-periosteal space. Nevertheless, careful surgical handling is required even during the retrieval of a Type 2 fragment; any inadvertent manipulation or mishandling can displace the fragment into deeper tissues, effectively converting the situation into a Type 3 scenario. A Type 3 scenario is significantly more challenging to manage due to limited surgical access, the proximity of critical structures such as the lingual nerve and submandibular gland, and a greater potential for complications during exploration and retrieval [2].

Surgical retrieval of a separated bur fragment located on the lingual aspect of the mandible is technically challenging due to several anatomical and clinical factors. These challenges include limited access and visibility, proximity to critical structures (particularly the lingual nerve and the inferior alveolar nerve), persistent bleeding and salivary flow in the area, and the potential for patient movement (tongue or jaw motion) during the procedure. Such complexities necessitate meticulous surgical technique, precise preoperative imaging localization, and cautious intraoperative handling of the fragment. In our case, the use of preoperative CBCT was crucial for planning the trajectory of access to the fragment and for anticipating its relationship to the lingual cortex and nerve. Similar cases documented in the literature illustrate a range of retrieval methods. Chen et al. reported a case in which computer-assisted navigation was employed to remove a bur fragment from the mandible, using a stereotactic reference frame attached to the jaw [15]. This high-tech approach allowed real-time guidance and minimized exploratory dissection. In contrast, Li et al. described a series of three cases where a more conventional technique was used: a straightforward approach involving soft tissue reflection and careful forceps retrieval under direct vision [16]. All three cases in their report were managed successfully without postoperative complications. Our approach aligned more closely with the latter, utilizing careful flap design and direct visualization aided by preoperative imaging, and resulted in a successful outcome with no adverse sequelae.

Another important consideration in our case was the choice of anesthesia for the fragment retrieval surgery. The decision between local anesthesia and general anesthesia (GA) depends on factors such as patient preference, the anticipated surgical difficulty, and the risks associated with each modality. While GA is often necessary for extensive or complex maxillofacial surgeries, local anesthesia is generally sufficient for minor to moderate procedures and carries a significantly lower risk profile. Common postoperative complaints associated with GA include sore throat and dysphagia (in up to 79% of cases), nausea (68%), vomiting (43%), pain and swelling (52%), headache (31%), and hypotension (30%) [17]. These side effects underscore that general anesthesia, although safe when properly administered, is not trivial and can contribute to patient morbidity. In contrast, local anesthesia in dentistry has a much lower incidence of complications. For example, transient paresthesia from local anesthetic injection occurs in approximately 0.15-0.54% of cases, and permanent nerve injury is exceedingly rare (estimated at 0.0001% and 0.01%) [18]. Allergic reactions to local anesthetics are also very uncommon (less than 1% of patients) [18]. Other potential complications of local anesthesia, such as trismus, infection, ocular complications, systemic toxicity, or methemoglobinemia, have been reported, but they are exceedingly rare and often lack well-established incidence rates [18]. Given these comparative risks and benefits, we proceeded with fragment removal under local anesthesia. This choice optimized patient safety, minimized the likelihood of anesthesia-related side effects, facilitated a faster recovery, and was well tolerated by the patient.

Successful management of retained surgical fragments like the one in this case relies on diligent follow-up and timely intervention when indicated. In our patient’s scenario, the fragment caused no immediate harm and was managed conservatively with observation until a change in circumstances (the need for MRI) mandated its removal. This highlights the importance of individualized patient management; continuous risk-benefit reassessment is necessary, as what is best for the patient may change over time. Early involvement of specialists, use of advanced imaging for planning, and patient education are all crucial in achieving a favorable outcome.

## Conclusions

Surgeons should always use sharp, suitable, and properly maintained surgical instruments, and avoid excessive force or heat during bone removal procedures to prevent complications such as instrument breakage. Regular inspection of instruments and prompt replacement of any dull or worn burs can significantly reduce the risk of such events. If an instrument fragment does separate during surgery, advanced imaging techniques like CBCT are invaluable for accurate localization of the fragment relative to critical anatomical structures. Prompt referral to an experienced oral and maxillofacial surgeon is recommended in these situations, and management should be guided by a thorough risk-benefit analysis tailored to the patient’s condition. Long-term clinical and radiographic monitoring is often necessary for retained fragments, particularly if the patient’s health status or diagnostic needs (such as an MRI requirement) change over time.

In the case presented, a delayed retrieval of the bur fragment under local anesthesia was performed safely after three years, with complete removal of the fragment and no permanent complications. This outcome underscores the importance of careful preoperative planning, precise surgical execution, and vigilant postoperative follow-up when managing retained surgical instrument fragments. By adhering to fundamental surgical principles and adapting the management strategy to the evolving clinical scenario, we achieved a successful resolution of an unusual complication.

## References

[1] Pitman KT (2009). Complications of surgery of the oropharynx. Complications in Head and Neck Surgery (Second Edition).

[2] Xing X, Gong C, Ye ZY, Lv K, Li Z (2025). Clinical characteristics and removal of broken burs retained in the lower jaw. BMC Oral Health.

[3] Chandak M, Agrawal P, Mankar N, Sarangi S, Bhopatkar J (2023). Navigating separated instrument retrieval: a case report. Cureus.

[4] Sacks GD, Dawes AJ, Ettner SL (2016). Surgeon perception of risk and benefit in the decision to operate. Ann Surg.

[5] DeSantana JM, Perissinotti DM, de Oliveira Jr JO, França Correia LM, de Oliveira CM, da Fonseca PR (2020). Definition of pain revised after four decades [Editorial]. BrJP.

[6] Barasuol JC, Santos PS, Moccelini BS (2020). Association between dental pain and oral health-related quality of life in children and adolescents: A systematic review and meta-analysis. Community Dent Oral Epidemiol.

[7] Matsuda S, Yoshimura H, Yoshida H, Sano K (2020). Breakage and migration of a high-speed dental hand-piece bur during mandibular third molar extraction: two case reports. Medicine (Baltimore).

[8] Pankhurst CL, Coulter WA (2017). Basic Guide to Infection Prevention and Control in Dentistry, 2nd Edition. https://www.wiley.com/en-us/Basic+Guide+to+Infection+Prevention+and+Control+in+Dentistry%2C+2nd+Edition-p-9781119164982#permissions-section.

[9] Savage NW, Walsh LJ (1995). The use of autoclaves in the dental surgery. Aust Dent J.

[10] Suhaimi FM, Roslan H, Musa Z, Azman N, Zukhi NJ (2018). The effect of multiple sterilisation cycles on cutting efficiency of a diamond bur. Malay J Med Health Sci.

[11] Andrabi SM, Alam S, Zia A, Khan MH, Kumar A (2014). Mental nerve paresthesia secondary to initiation of endodontic therapy: a case report. Restor Dent Endod.

[12] Chhabra A, Ahlawat S, Belzberg A, Andreseik G (2014). Peripheral nerve injury grading simplified on MR neurography: as referenced to Seddon and Sunderland classifications. Indian J Radiol Imaging.

[13] Peschke E, Ulloa P, Jansen O, Hoevener JB (2021). Metallic implants in MRI - hazards and imaging artifacts. Rofo.

[14] Jungmann PM, Agten CA, Pfirrmann CW, Sutter R (2017). Advances in MRI around metal. J Magn Reson Imaging.

[15] Chen S, Liu YH, Gao X, Yang CY, Li Z (2020). Computer-assisted navigation for removal of the foreign body in the lower jaw with a mandible reference frame: a case report. Medicine (Baltimore).

[16] Li K, Xie B, Chen J, He Y (2022). Breakage and displacement of the high-speed hand-piece bur during impacted mandibular third molar extraction: three cases. BMC Oral Health.

[17] Saodekar R, Gondhalekar R, Kalamkar J (2022). Common postoperative complications following general anesthesia in oral and maxillofacial surgery: a cross-sectional study. Acad Anesthesiol.

[18] Cummings DR, Yamashita DD, McAndrews JP (2011). Complications of local anesthesia used in oral and maxillofacial surgery. Oral Maxillofac Surg Clin North Am.

